# Comparative effects of corn, wheat, and barley diets on broiler growth performance, carcass traits, meat quality, and consumer sensory evaluation

**DOI:** 10.14202/vetworld.2025.4117-4128

**Published:** 2025-12-27

**Authors:** Patcharawan Kongkasem, Choawit Rakangthong, Phongthorn Kongman, Sombat Prasongsook, Kasama Sudtilak, Theerawit Poeikhampha

**Affiliations:** Department of Animal Science, Faculty of Agriculture, Kasetsart University, Bangkok, Thailand

**Keywords:** broiler, growth performance, carcass quality, meat quality, consumer sensory evaluation, corn, wheat, barley

## Abstract

**Background and Aim:**

Corn is the main cereal used in broiler nutrition because of its high energy content and carotenoid richness, while wheat and barley offer alternative nutrient profiles that may increase production flexibility. However, their relative impacts on broiler performance, carcass traits, meat physicochemical properties, and consumer sensory perception under standardized enzyme-supplemented conditions remain unclear. This study examined the effects of partially replacing corn with wheat or barley on growth performance, carcass yield, meat quality, and consumer sensory evaluation in broiler chickens.

**Materials and Methods:**

A total of 525 male ROSS 308 broilers were randomly assigned to three dietary treatments: corn (C), corn–wheat (CW), and corn–barley (CB), with five replicates of 35 birds each. Diets were isocaloric and isonitrogenous and supplemented with a xylanase–β-glucanase complex. Birds were raised for 35 days under controlled environmental conditions. Growth performance, carcass traits, meat color (L*, a*, b*), pH, water-holding capacity, texture profile, and consumer sensory attributes were evaluated using standardized protocols. Data were analyzed using General Linear Model procedures with significance set at p < 0.05.

**Results:**

Broilers fed with corn showed numerically higher body weight gain (+4.8% compared to CW; +4.4% compared to CB) and a tendency toward improved feed conversion ratio (1.52 vs. 1.56–1.58; p = 0.10). Including barley significantly increased abdominal fat (+36% vs. corn; p = 0.04), while wheat resulted in the lowest fat deposition. Meat yellowness (b*) was highest in the corn group at both 45 min and 24 h postmortem (p < 0.05, p < 0.01), reflecting the higher carotenoid content of corn. No significant differences were found among treatments for pH, drip loss, cooking and thawing loss, texture parameters, or sensory scores (p > 0.05). All sensory attributes scored above 4.3 on the 7-point scale.

**Conclusion:**

Moderate inclusion of wheat (12%–20%) or barley (8%–15%) in enzyme-supplemented diets did not affect growth performance, carcass yield, meat physicochemical traits, or consumer sensory acceptance. Wheat might be used strategically to reduce abdominal fat, while corn remains preferred when enhanced yellowness is desired. These findings support the practical use of wheat and barley as viable alternatives to corn in commercial broiler feeding programs.

## INTRODUCTION

Carcass and meat quality are key factors in the profitability of broiler production, as they directly impact processing efficiency, product grading, and consumer preferences. Important carcass traits, such as breast, thigh, and wing yields, along with key meat qualities like tenderness, juiciness, and color, significantly affect the marketability of broiler products [1, 2]. Cereal grains form the main energy source in broiler diets, with corn (*Zea mays*), wheat (*Triticum aestivum*), and barley (*Hordeum vulgare*) being the most common ingredients. Corn is traditionally preferred due to its high metabolizable energy and low non-starch polysaccharide (NSP) content [3]. In contrast, wheat offers a more balanced protein-to-energy ratio and can improve gut development when supplemented with carbohydrase enzymes [4]. Barley, although used less often due to its high β-glucan content, which increases intestinal viscosity and reduces nutrient digestibility, provides functional benefits through its soluble fiber and bioactive compounds that support intestinal health [5–7]. Recent advances in exogenous enzyme technologies have further enhanced nutrient utilization from NSP-rich cereals, enabling broader use of wheat and barley in modern broiler feeding programs.

The intrinsic composition of these cereals differently influences growth performance, carcass traits, and meat quality outcomes. Corn’s naturally high carotenoid content, especially xanthophylls, contributes to increased yellowness in skin and meat, which is desirable in several Asian and Latin American markets [8, 9]. Wheat and barley, with lower carotenoid levels, generally produce paler carcasses; however, their higher NSP levels may decrease abdominal fat deposition by altering energy partitioning and increasing digesta viscosity [10]. Although wheat-based diets typically improve nutrient digestibility when supplemented with appropriate enzymes [4], including barley without sufficient fibrolytic enzyme support can impair growth due to NSP-related anti-nutritive effects. Nonetheless, emerging evidence suggests that barley diets enhanced with xylanase–β-glucanase complexes can achieve performance levels similar to those of corn-based formulations [6, 7].

Despite the widespread use of corn, wheat, and barley in poultry diets, the existing research remains fragmented regarding their direct comparative effects on broiler performance, carcass traits, and meat quality when evaluated under fully standardized nutritional conditions. Most previous studies have examined these cereals separately, used varying inclusion levels, or lacked isocaloric, isonitrogenous diets, making cross-study comparisons difficult. Additionally, differences in experimental conditions, such as broiler genotype, enzyme supplementation, processing methods, or environmental stressors, further complicate the interpretation of outcomes related to growth, carcass yield, and meat physicochemical traits. Moreover, although wheat and barley are increasingly seen as alternative energy sources in regions where corn supply fluctuates, limited research has incorporated consumer sensory evaluations alongside physicochemical meat quality, despite their importance for market acceptance. Therefore, a clear, integrated understanding of how these cereals influence both production efficiency and consumer-perceived product quality is still lacking, especially under controlled conditions that replicate commercial feed practices.

To address this knowledge gap, the present study aimed to systematically evaluate the effects of partially replacing corn with wheat or barley on growth performance, carcass yield, meat physicochemical properties, and consumer sensory perception in broiler chickens. Using isocaloric and isonitrogenous diets supplemented with a standardized xylanase–β-glucanase enzyme complex, this study sought to isolate the influence of cereal type from confounding nutritional factors. The controlled design allowed for the assessment of cereal-specific effects on body weight gain (BWG), feed efficiency, carcass fat deposition, muscle color, pH, water-holding capacity, texture traits, and consumer acceptance of cooked meat. By combining performance metrics, carcass data, laboratory meat quality evaluations, and sensory responses within a single experimental framework, this study aimed to provide comprehensive, evidence-based insights supporting more flexible and sustainable cereal sourcing strategies in commercial broiler production.

## MATERIALS AND METHODS

### Ethical approval

All experimental procedures involving animals were performed following the institutional guidelines for animal care and use in research. The study protocol was reviewed and approved by the Animal Care and Use Committee of Kasetsart University, Thailand (Approval No. ACKU64-AGR-015). All husbandry, handling, vaccination, and slaughter procedures adhered to accepted animal welfare standards to reduce stress and ensure ethical treatment throughout the study.

The sensory evaluation involved minimally processed, commercially safe cooked poultry meat and posed no risks to participants. All sensory panelists were adults who voluntarily participated and provided informed consent prior to involvement. No personal identifiers were collected, and anonymity was maintained throughout the data collection process. Because the activity involved minimal risk, no invasive procedures, and evaluation of fully cooked food products, the sensory evaluation did not require formal approval under the institutional human research ethics framework.

### Study period and location

This study was conducted from July 2021 to September 2021 at the Animal Research Farm and Animal Feed Analysis Laboratory, Department of Animal Science, Faculty of Agriculture, Kasetsart University, Thailand.

### Experimental animals and their housing

The experiment involved 525 one-day-old male broiler chickens (ROSS 308) conducted under a completely randomized design. Three dietary treatments (corn-based diet: C; corn–wheat diet: CW; and corn–barley diet: CB) were tested, with five replicates per treatment and 35 birds in each pen, totaling 15 pens. Birds were randomly assigned to treatments using a computer-generated list to ensure unbiased distribution. They were raised on the floor in a closed-house system with an evaporative cooling system and tunnel-ventilation, powered by four exhaust fans at the end. The concrete floor was covered with rice husk litter about 5 cm deep. Stocking density was maintained at 11.67 birds/m², with ad libitum access to feed and water throughout the study. The water met standard farm-quality criteria, with a pH of 6.8–7.2 and water hardness between 40 and 60 ppm, supplied from a treated groundwater system and available at nipple drinkers. The rice husk litter was manually stirred every 2–3 days to keep it dry and limit ammonia buildup.

From day 1 to 10, each pen was equipped with a brooder lamp for chicks; feed was provided in chick feeders, and water was supplied in chick drinkers. From days 11 to 35, feed was offered in suspended feeders (10-inch diameter), and water was supplied via an automatic nipple drinker system with seven nipples per pen. On day 1, all birds were vaccinated against Newcastle disease, infectious bursal disease (Gumboro), and infectious bronchitis. On day 10, they were vaccinated against Newcastle disease and infectious bronchitis.

### Environmental conditions

The birds were raised under controlled environmental conditions throughout the 35-day experimental period. The ambient temperature gradually decreased from an average of 32.9°C on day 1 to 28.9°C by day 35, with daily minimum and maximum temperatures ranging between 26.7°C and 37.6°C. The relative humidity fluctuated between 47.7% and 89.5%, averaging 60%–70% during the starter phase and increasing to approximately 78%–82% during the finisher phase. These conditions remained within acceptable limits for commercial broiler production and were managed with an evaporative cooling and tunnel-ventilation system to ensure consistent airflow and thermal comfort. All pens were monitored daily to maintain uniform temperature, humidity, and air quality across all treatments.

### Experimental diets

The experimental diets were formulated according to Aviagen’s nutritional requirements for ROSS 308 broilers [11] and contained no antibiotic growth promoters. Feeds were prepared in a crumble form during the starter period (1–7 days) and pelleted afterward (8–35 days). All ingredients were ground with a 3-mm hammer mill screen, mixed for 15 min, and conditioned with steam at approximately 80°C before pelleting. The oil and liquid additives were added during the final mixing stage. The finished pellets were cooled to approximately 25°C–30°C (room temperature) and stored in a dry, protected environment until feeding.

All cereal ingredients (corn, wheat, and barley) were obtained as standard commercial feed-grade grains from a certified feed mill, without specifying their botanical varieties. This reflects typical raw material sourcing practices in commercial broiler production. All raw materials were routinely screened for quality by the feed mill, including checks for moisture content, mycotoxin contamination, and general antinutritional factors, following standard commercial feed safety procedures. The cereals were formulated using standardized nutrient matrices commonly used in commercial feed mill quality control programs to ensure consistency and nutritional accuracy.

Three dietary treatments were formulated: C, CW, and CB. In the CW and CB diets, corn was partially replaced with wheat (12%–20%) or barley (8%–15%), depending on the feeding phase, with inclusion levels adjusted to maintain isocaloric and isonitrogenous formulations across treatments. The feeding program included a starter phase (1–10 days), a grower phase (11–24 days), and a finisher phase (25–35 days). Because the metabolizable energy content varies among these cereals, the inclusion levels of soybean oil were adjusted to keep diets isocaloric. All diets were created to be both isocaloric and isonitrogenous, following Aviagen nutrient guidelines.

NSP-degrading enzymes were added to all diets at a rate of 0.01% (100 g/ton). The enzyme was a commercial xylanase–β-glucanase blend commonly used in broiler feed formulations during the study, providing about 1,500 U of xylanase/kg and 200 U of β-glucanase/kg of finished feed. The enzyme product was obtained from a certified commercial feed additive supplier regularly used in broiler feed production and was added according to the manufacturer’s recommended rate. This enzyme combination was chosen because xylanase and β-glucanase are widely used in wheat- and barley-based broiler diets to enhance nutrient digestibility and reduce digesta viscosity.

Nutrient compositions were determined using standardized ingredient matrices from a certified commercial feed mill. No post-formulation proximate or amino acid analyses were conducted because the mill follows routine quality assurance procedures with validated nutrient specifications. Table 1 shows the ingredient composition of each diet, and their respective calculated nutrient compositions are listed in Table 2.

**Table 1:** Composition of the ingredients of the experimental diets.

Items	Starter (1–10 days)			Grower (11–24 days)			Finisher (25–35 days)		
		
	C	CW	CB	C	CW	CB	C	CW	CB
Corn	32.94	21.41	24.43	35.96	16.51	22.99	40.00	22.10	26.60
Cassava	20.00	20.00	20.00	20.00	20.00	20.00	20.00	20.00	20.00
Wheat	-	12.00	-	-	20.00	-	-	20.00	-
Barley	-	-	8.00	-	-	12.00	-	-	15.00
Soybean oil	1.50	1.97	2.36	2.40	3.33	3.82	3.00	3.00	3.00
Soybean 48% CP	40.21	39.25	39.85	36.85	35.34	36.38	32.51	30.50	30.98
DL-Methionine	0.40	0.40	0.40	0.35	0.34	0.35	0.32	0.31	0.32
L-Lysine	0.21	0.23	0.21	0.14	0.16	0.13	0.13	0.16	0.14
L-Threonine	0.11	0.12	0.11	0.07	0.08	0.07	0.04	0.06	0.05
L-Valine	0.03	0.03	0.02	-	-	-	-	-	-
CaCo_3_ (Limestone)	1.12	1.12	1.11	1.01	1.01	1.00	0.91	0.92	0.91
Monocalcium phosphate (21% P)	2.13	2.12	2.14	1.89	1.88	1.91	1.70	1.68	1.72
Salt	0.32	0.32	0.32	0.32	0.32	0.32	0.33	0.32	0.32
60% choline chloride	0.28	0.28	0.28	0.27	0.27	0.27	0.25	0.25	0.25
Vitamin PX	0.33	0.33	0.33	0.33	0.33	0.33	0.33	0.33	0.33
Mineral PX	0.33	0.33	0.33	0.33	0.33	0.33	0.33	0.33	0.33
Mold inhibitor	0.05	0.05	0.05	0.05	0.05	0.05	0.05	0.05	0.05
Antioxidant	0.05	0.05	0.05	0.05	0.05	0.05	0.01	0.01	0.01
NSP Enz.	0.01	0.01	0.01	0.01	0.01	0.01	0.01	0.01	0.01

C = Corn group, CW = Corn + Wheat group, CB = Corn + Barley group, CP = Crude protein, CaCo3 = Calcium carbonate, P = Phosphorus, NSP Enz. = Commercial non-starch polysaccharide–degrading enzyme complex (xylanase + β-glucanase), included at 0.01% (100 g/ton), providing ~1,500 U xylanase/kg and ~200 U β-glucanase/kg of feed. The enzyme was added to reduce digesta viscosity and improve nutrient digestibility in wheat- and barley-based diets.

**Table 2:** Nutrient composition of the experimental diets.

Items	Starter (1-10 d)			Grower (11-24 d)			Finisher (25-35 d)		
		
	C	CW	CB	C	CW	CB	C	CW	CB
ME for poultry (kcal/kg)	3,000.00	3,000.00	3,000.00	3,100.00	3,100.00	3,100.00	3,200.00	3,200.00	3,200.00
Crude protein (%)	23.00	23.00	23.00	21.50	21.50	21.50	19.50	19.50	19.50
Fat (%)	3.38	3.62	4.07	4.34	4.88	5.49	4.93	4.64	4.77
Crude fiber (%)	2.98	3.03	3.16	2.90	2.97	3.15	2.74	2.84	3.10
Calcium (%)	0.96	0.96	0.96	0.87	0.87	0.87	0.79	0.79	0.79
Total P (%)	0.81	0.81	0.81	0.74	0.75	0.74	0.68	0.69	0.68
Avail. P (%)	0.48	0.48	0.48	0.44	0.44	0.44	0.40	0.40	0.40
Salt (%)	0.39	0.39	0.39	0.39	0.39	0.39	0.39	0.39	0.39
Lysine (%)	1.44	1.44	1.44	1.29	1.29	1.29	1.16	1.16	1.16
Methionine (%)	0.72	0.72	0.72	0.65	0.64	0.65	0.60	0.59	0.59
Met + cystine (%)	1.08	1.08	1.08	0.99	0.99	0.99	0.91	0.91	0.91
Tryptophan (%)	0.28	0.29	0.29	0.27	0.27	0.27	0.24	0.24	0.24
Threonine (%)	0.97	0.97	0.97	0.88	0.88	0.88	0.78	0.78	0.78
Arginine (%)	1.56	1.55	1.56	1.45	1.45	1.46	1.31	1.29	1.30
Valine (%)	1.10	1.10	1.10	1.01	1.01	1.01	0.92	0.91	0.92
Isoleucine (%)	0.99	0.99	1.00	0.93	0.93	0.94	0.84	0.84	0.84
Leucine (%)	1.82	1.77	1.79	1.73	1.65	1.68	1.59	1.52	1.55
Choline (mg/kg)	1,700.00	1,700.00	1,700.00	1,600.00	1,600.00	1,600.00	1,500.00	1,500.00	1,500.00

C = Corn group, CW = Corn + Wheat group, CB = Corn + Barley group, ME = Metabolizable energy, P = Phosphorus.

### Growth performance measurements

Growth performance was assessed over four time periods: 1–10, 11–24, 25–35, and 1–35 days. Individual body weight (BW) was recorded at the start of each period, and feed intake was measured at the pen level. The average daily gain (ADG), average daily feed intake (ADFI), feed conversion ratio (FCR), feed cost per gain (FCG) = FCR × feed cost, and mortality were calculated. The dietary cost per kilogram was determined using ingredient prices from the commercial feed mill during the study period. To reduce diurnal variation, all measurements were taken at the same time each day, and digital scales were calibrated before each weighing session.

### Carcass quality determination

On day 36, three birds per pen (n = 15 per treatment) were selected based on the pen average BW (±5% of mean BW) using a random number generator. The birds underwent a standardized 4 h feed withdrawal and were euthanized by CO_2_ asphyxiation (<6% O_2_), followed by exsanguination and defeathering under identical processing conditions. Carcasses were dissected following standardized anatomical landmarks to obtain the weights of the outer breast, inner breast, wing, thigh, drumstick, and abdominal fat, as described by Hashim *et al*. [12]. To ensure consistency across samples, the Pectoralis major muscle was removed for all subsequent meat quality analyses.

### Meat quality assessments

#### Meat color and pH measurements

Three chickens per pen (the same animals used for carcass evaluation) were selected based on their average weight and slaughtered following the standardized protocol described above. The pH of the outer breast muscle was measured at 45 min and 24 h post-slaughter using a calibrated pH meter. Before each measurement, calibration was performed with pH 4.0 and 7.0 buffer solutions. Meat color was assessed on the medial surface of the breast using a Minolta chroma meter, recording values for lightness (L*), redness (a*), and yellowness (b*). All measurements were conducted in triplicate at each time point following standard procedures [13].

#### Water-holding capacity

Raw breast meat samples were individually packed in plastic bags and stored at 4°C for 24 h to determine drip loss. The thawing loss was assessed after storage at −20°C, followed by thawing at 4°C for 24 h before measurement. Cooking loss was measured by cooking samples in a water bath at 80°C until reaching an internal temperature of 75°C, then cooling to room temperature before final weighing. All procedures were performed using calibrated digital scales and followed the standard analytical methods described by Caldas-Cueva *et al*. [13]. The procedures for measuring drip loss, thawing loss, and cooking loss were adapted from the general guidelines of the Association of Official Analytical Collaboration (AOAC) Method 983.18, a standardized method for determining moisture content in animal feeds and pet food. This method incorporated fixed storage at 24 h, controlled thawing conditions, and a water bath set to 80°C to achieve the target internal temperature recommended for evaluating poultry meat.

#### Texture profile analysis

To ensure consistent sample preparation, texture profile measurements were performed on cooked Pectoralis major samples obtained after determining cooking loss. For Warner–Bratzler shear force analysis, breast muscle strips (1 cm height × 1 cm width × 2.5 cm length) were tested using a Warner–Bratzler blade attached to a TA-XT Texture Analyzer (Model TA-XT Plus; Stable Micro Systems, Godalming, Surrey, UK). Each measurement was conducted in triplicate following the procedures described by Chatterjee *et al*. [14]. Texture profile analysis was carried out on cooked, skinless breast meat cores (1 cm height × 1 cm diameter) using the same texture analyzer according to established methods [15]. The parameters measured included hardness, cohesiveness, springiness, gumminess, and chewiness.

### Consumer sensory evaluation

The skinless breast meat samples were vacuum-cooked at 80°C for 20 min and cut into uniform cubes (1 × 1 × 1 cm). Sensory evaluation was performed using a panel of 30 randomly recruited volunteer consumers, representing typical broiler meat consumers. All panelists were untrained and selected based on willingness to participate, regular poultry consumption, and absence of food allergies. Each sample was labeled with a randomized three-digit code to ensure blinding, and the serving order was randomized and counterbalanced across participants to prevent positional bias. Each panelist received 25 g of each sample in identical single-use containers and evaluated the samples in duplicate. To minimize carry-over effects, unsweetened tea was provided between samples. The experiments were conducted under controlled environmental conditions (22°C ± 1°C, neutral lighting). Sensory attributes, including appearance, color, texture, tenderness, odor, taste, and overall acceptance, were scored using a 0–7 intensity scale following the quantitative descriptive analysis method described by De Pilli *et al*. [16].

### Statistical analysis

Data were analyzed using the General Linear Model procedure of SAS® OnDemand for Academics (SAS Institute Inc., Cary, NC, USA) following the SAS/STAT® guidelines [17]. Each pen was considered the experimental unit for all performance, carcass, meat quality, texture, and sensory variables. Before conducting analysis of variance, the data were checked for normality with the Shapiro–Wilk test and for homogeneity of variances using Levene’s test. When assumptions were not met, the data were log-transformed and reanalyzed; however, the interpretation remained the same, and the results were presented on the original scale.

The statistical model used in the analysis was as follows:

*Y_ij_*=*μ+T_i_*+*ε_ij_*

where *Y_ij_* represents the observation from pen j assigned to treatment i, *μ* is the overall mean, *T_i_* is the fixed effect of dietary treatment, and *ε_ij_* is the random error term.

The mean sensory scores per treatment were calculated by averaging the duplicate evaluations from all panelists before statistical analysis. Data were also screened for outliers using studentized residuals; however, no extreme values warranted removal. All analyses were conducted on the cloud-based SAS® OnDemand for Academics platform. When significant treatment effects were found (p < 0.05), Duncan’s multiple range test was used to compare means, while values between 0.05 and 0.10 were considered trends. All results are presented as means ± standard error of the mean.

## RESULTS

### Growth performance

Table 3 summarizes the growth performance of broilers fed diets containing corn (C), corn–wheat (CW), or corn–barley (CB) across different production phases. Wheat or barley inclusion had no significant effect on initial BW, ADFI, FCG, or mortality across all phases (p > 0.05). Most mortality occurred during the first week and was primarily attributed to early chick weakness rather than treatment-related or infectious causes, as no clinical signs or pathological lesions indicative of disease were observed. During the grower phase, corn-fed broilers tended to have higher final BW and BWG (p = 0.06, p = 0.07). In the finisher phase, the corn group also showed numerically higher final BW and BWG than the wheat and barley groups (p = 0.08 and 0.37, respectively). Although differences in the FCR were not significant, corn-fed broilers showed a tendency toward improved efficiency (lower FCR), while the CB group exhibited slightly less favorable values (p = 0.10).

**Table 3:** Effect of feed energy source components on the growth performance of broiler chickens (1-35 days).

Items	C	CW	CB	SEM	p-value
Starter broilers (1–10 days)					
Initial BW (g)	43.25 ± 0.54	43.25 ± 0.58	43.26 ± 0.47	0.13	0.99
Final BW (g)	252.57 ± 6.72	254.49 ± 9.07	253.86 ± 4.15	1.67	0.91
BWG (g)	209.32 ± 7.07	211.24 ± 9.49	210.60 ± 4.30	1.75	0.91
ADFI (g)	25.38 ± 0.71	25.57 ± 2.52	26.94 ± 0.57	0.41	0.26
FCR	1.21 ± 0.06	1.21 ± 0.13	1.28 ± 0.04	0.02	0.37
FCG (baht/kg)	16.31 ± 0.90	16.23 ± 1.74	17.29 ± 0.49	0.31	0.31
Mortality (%)	0.57 ± 1.28	0.00 ± 0.00	0.00 ± 0.00	0.19	0.40
Grower broilers (11–24 days)					
Initial BW (g)	254.08 ± 8.18	256.58 ± 10.80	253.86 ± 4.15	1.98	0.85
Final BW (g)	1308.52 ± 24.04	1262.35 ± 18.49	1263.98 ± 44.90	9.41	0.06
BWG (g)	1054.44 ± 26.76	1005.76 ± 27.69	1010.12 ± 42.45	9.85	0.07
ADFI (g)	91.39 ± 1.68	87.69 ± 1.18	89.13 ± 4.94	0.84	0.20
FCR	1.21 ± 0.03	1.22 ± 0.04	1.24 ± 0.04	0.01	0.63
FCG (baht/kg)	16.09 ± 0.52	16.31 ± 0.64	16.55 ± 0.48	0.14	0.44
Mortality (%)	0.63 ± 1.40	0.65 ± 1.44	0.00 ± 0.00	0.29	0.62
Finisher broilers (25–35 days)					
Initial BW (g)	1308.52 ± 24.04	1262.35 ± 18.49	1263.98 ± 44.90	9.41	0.06
Final BW (g)	2248.02 ± 102.70	2136.33 ± 57.47	2150.23 ± 59.71	22.54	0.08
BWG (g)	939.50 ± 100.94	873.98 ± 60.39	886.25 ± 56.29	19.54	0.37
ADFI (g)	145.00 ± 9.37	139.65 ± 7.18	141.55 ± 5.23	1.88	0.53
FCR	1.70 ± 0.10	1.76 ± 0.07	1.76 ± 0.09	0.02	0.53
FCG (baht/kg)	21.90 ± 1.40	22.07 ± 0.86	22.28 ± 1.41	0.30	0.89
Mortality (%)	1.23 ± 1.69	0.00 ± 0.00	0.65 ± 1.44	0.33	0.35
Total broiler period (1–35 days)					
Initial BW (g)	43.25 ± 0.54	43.25 ± 0.58	43.26 ± 0.47	0.13	0.99
Final BW (g)	2248.02 ± 102.70	2136.33 ± 57.47	2150.23 ± 59.71	22.54	0.08
BWG (g)	2204.77 ± 102.93	2093.08 ± 57.76	2106.97 ± 60.15	22.60	0.08
ADFI (g)	89.38 ± 3.56	86.27 ± 2.33	87.84 ± 3.45	0.83	0.33
FCR	1.42 ± 0.02	1.44 ± 0.03	1.46 ± 0.03	0.01	0.10
FCG (baht/kg)	18.83 ± 0.54	18.75 ± 0.51	19.08 ± 0.65	0.14	0.64
Mortality (%)	2.43 ± 2.51	0.65 ± 1.44	0.65 ± 1.44	0.50	0.26

C = Corn group, CW = Corn + Wheat group, CB = Corn + Barley group, SEM = Standard error of the mean, BW = Body weight, BWG = Body weight gain, ADFI = Average daily feed intake, FCR = Feed conversion ratio, FCG = Feed cost per kg gain.

### Carcass quality

As shown in Table 4, aside from abdominal fat, replacing corn with wheat or barley did not significantly impact carcass yield or the proportion of individual parts (p > 0.05). Broilers in the CB group had the highest abdominal fat percentage, while those in the corn group had the lowest (p = 0.04). The proportions of wings, inner breast, outer breast, drumstick, and thighs were consistent across different treatments.

**Table 4:** Effect of feed energy source components on the carcass quality of broiler chickens at 36 days of age.

Items	C	CW	CB	SEM	p-value
%Carcass	83.16 ± 1.93	83.02 ± 2.04	83.86 ± 2.15	0.37	0.62
%Wing	8.90 ± 0.34	9.30 ± 0.44	9.25 ± 0.51	0.08	0.10
%Inner breast	4.35 ± 0.53	4.28 ± 0.35	4.45 ± 0.44	0.08	0.71
%Outer breast	25.21 ± 1.90	24.93 ± 2.06	25.65 ± 2.05	0.36	0.72
%Drumstick	12.62 ± 0.84	12.31 ± 0.66	12.55 ± 1.23	0.17	0.75
%Thigh	16.29 ± 1.46	15.69 ± 1.56	15.51 ± 1.49	0.27	0.48
%Abdominal fat	1.02 ± 0.36^b^	1.27 ± 0.31^ab^	1.39 ± 0.27^a^	0.06	0.04

C = Corn group, CW = Corn + Wheat group, CB = Corn + Barley group, SEM = Standard error of the mean. ^a,b^ Means in the same row with different superscripts differ significantly (p < 0.05).

### Meat quality

#### Physicochemical properties

The physicochemical properties of the meat are shown in Table 5. Replacing corn with wheat or barley had no significant impact on pH, drip loss, cooking loss, or thawing loss at 45 min or 24 h post-slaughter (p > 0.05). However, there were notable differences in meat yellowness (b*). At 45 min, corn-fed broilers showed the highest yellowness values, while the CW group had the lowest (p < 0.05). At 24 hours, yellowness remained highest in the corn group, with both wheat and barley groups exhibiting lower values (p < 0.01). Lightness (L*) and redness (a*) showed no significant differences among treatments at either time point.

**Table 5:** Effect of energy source components in feed on broiler chicken meat quality and water-holding capacity.

Items	C	CW	CB	SEM	p-value
pH 45 min	6.38 ± 0.22	6.37 ± 0.12	6.34 ± 0.18	0.03	0.88
pH 24 h	5.87 ± 0.13	5.79 ± 0.13	5.82 ± 0.13	0.03	0.47
Drip loss (%)	2.58 ± 0.51	2.36 ± 0.36	2.54 ± 0.62	0.10	0.65
Cooking loss (%)	26.15 ± 3.87	26.45 ± 5.29	24.63 ± 2.33	0.80	0.63
Thawing loss (%)	4.56 ± 2.98	3.51 ± 2.06	3.15 ± 1.50	0.46	0.45
Color 45 min					
Lightness (L*)	51.25 ± 4.76	47.88 ± 5.78	50.76 ± 5.11	1.07	0.40
Redness (a*)	3.10 ± 1.18	3.41 ± 2.68	2.87 ± 1.07	0.35	0.84
Yellowness (b*)	6.14 ± 2.10^a^	3.46 ± 1.20^b^	4.80 ± 1.68^ab^	0.40	0.02
Color 24 h					
Lightness (L*)	56.13 ± 2.57	55.43 ± 2.90	55.27 ± 2.64	0.53	0.80
Redness (a*)	4.51 ± 1.08	3.98 ± 0.81	4.01 ± 1.08	0.20	0.50
Yellowness (b*)	8.19 ± 1.06^A^	5.75 ± 1.50^B^	6.56 ± 1.13^B^	0.32	<0.01

C = Corn group, CW = Corn + Wheat group, CB = Corn + Barley group. ^a, b^ Means in the same row with different superscripts differ significantly (p < 0.05). ^A, B^ Means in the same row with different superscripts differ significantly (p < 0.01).

#### Texture profile

Table 6 presents the texture parameters. No significant differences were found in shear force, hardness, adhesiveness, springiness, cohesiveness, gumminess, or chewiness across dietary treatments (p > 0.05). All texture attribute values fell within the normal range for breast meat.

**Table 6:** Effect of feed energy source components on the meat texture profile of broiler chickens.

Items	C	CW	CB	SEM	p-value
Shear force (N)	21.63 ± 6.44	19.57 ± 4.99	18.47 ± 3.93	1.06	0.48
Hardness (N)	24.62 ± 7.38	22.20 ± 5.71	21.10 ± 4.46	1.20	0.49
Adhesiveness	8.03 ± 2.75	7.40 ± 2.15	7.42 ± 4.56	0.65	0.91
Springiness	0.61 ± 0.03	0.59 ± 0.03	0.59 ± 0.03	0.01	0.21
Cohesiveness	0.54 ± 0.03	0.55 ± 0.06	0.52 ± 0.02	0.01	0.39
Gumminess	13.59 ± 4.29	12.47 ± 3.93	11.04 ± 2.18	0.73	0.38
Chewiness	8.49 ± 3.06	7.43 ± 2.49	6.52 ± 1.48	0.50	0.29

C = Corn group, CW = Corn + Wheat group, CB = Corn + Barley group

### Consumer sensory evaluation

Table 7 shows the sensory evaluation scores for appearance, color, odor, texture, tenderness, taste, and overall acceptance. No significant differences were found among the treatments (p > 0.05). Mean scores for all attributes were above 4.3 on the 7-point scale, indicating positive consumer perception across all dietary treatments.

**Table 7:** Effect of energy source components in feed on the consumer sensory evaluation score of broiler chicken meat.

Items	C	CW	CB	SEM	p-value
Appearance	4.56 ± 1.15	4.63 ± 1.18	4.38 ± 1.06	0.13	0.72
Color	4.68 ± 1.09	4.61 ± 1.26	4.56 ± 1.01	0.12	0.92
Odor	3.90 ± 1.40	4.31 ± 1.42	4.36 ± 1.34	0.15	0.39
Texture	4.45 ± 1.30	4.79 ± 1.23	4.75 ± 0.97	0.13	0.49
Tenderness	4.28 ± 1.39	4.62 ± 1.37	4.54 ± 1.17	0.14	0.58
Taste	4.31 ± 1.23	4.62 ± 1.45	4.86 ± 1.01	0.13	0.26
Acceptance	4.50 ± 1.20	5.00 ± 1.05	4.83 ± 0.89	0.11	0.18

C = Corn group, CW = Corn + Wheat group, CB = Corn + Barley group, SEM = Standard error of the mean.

## DISCUSSION

### Growth performance

In the present study, broilers fed corn-based diets tended to have higher final BW and BWG during the grower and finisher phases than those fed wheat- or barley-based diets, despite all diets being isocaloric and supplemented with NSP-degrading enzymes. In the grower phase, corn-fed broilers had a 4.8% higher BWG than wheat-fed birds (p = 0.07) and 4.4% higher than barley-fed birds (p = 0.07). This trend is consistent with corn’s superior nutrient digestibility and lower NSP content, which reduces intestinal viscosity and enhances nutrient absorption [7, 18]. Enzyme supplementation improves digestibility in wheat and barley diets [19, 20], whereas low NSP levels in corn make it less dependent on enzymatic aid for optimal performance [21, 22].

### Carcass quality

Replacing corn with barley notably increased abdominal fat percentage, with barley-fed broilers showing a 36% higher value than corn-fed birds (p = 0.04). Conversely, including wheat resulted in the lowest abdominal fat percentage. Differences in starch digestibility and fiber content can explain these variations. Corn, with its high starch (65%–70%) and low fiber levels, offers easily digestible energy that may promote lipid deposition [23]. The higher NSP content of wheat may decrease energy absorption and fat storage [10, 24]. In barley, the presence of β-glucans raises gut viscosity, reducing nutrient absorption and potentially affecting lipid metabolism [25, 26]. This indicates that partial wheat inclusion could be a strategy to decrease carcass fat without reducing yield for poultry producers.

### Meat color and physicochemical traits

Significant differences were observed in meat yellowness (b*), with corn-fed broilers showing the highest values both at 45 min and 24 h post-slaughter (p < 0.05, p < 0.01). This aligns with the high carotenoid content of corn, especially xanthophylls, which are deposited in muscle and fat tissues [9] and may affect pigment-related gene expression [8]. Wheat and barley, which contain lower levels of carotenoids, resulted in paler meat color, which might be less appealing in markets that favor yellowish meat. Nonetheless, no differences were found in pH, drip loss, cooking loss, thawing loss, or texture profile, indicating that grain type does not compromise physicochemical meat quality when diets are balanced and include enzyme supplements [19, 27].

### Consumer sensory evaluation

Despite differences in meat yellowness and abdominal fat content, there were no significant differences in sensory scores for appearance, color, odor, texture, tenderness, taste, and overall acceptance among treatments (p > 0.05). All groups achieved mean scores above 4.3 on a 7-point scale, indicating generally favorable consumer perceptions. This aligns with previous findings that consumers may prioritize tenderness, juiciness, and flavor over visual color differences when evaluating cooked meat [28, 29]. For the poultry industry, this suggests that alternative cereals, such as wheat and barley, can replace corn without compromising consumer sensory evaluation, provided formulations are optimized.

### Role of enzyme supplementation

Enzyme supplementation likely played a key role in reducing the antinutritive effects of wheat and barley. Xylanase and β-glucanase are known to lower digesta viscosity, increase nutrient availability, and enhance metabolizable energy in high-NSP cereals, which helps restore performance to levels similar to corn-based diets. From a sustainability perspective, the ability to include wheat and barley without negatively affecting carcass yield, meat quality, or consumer acceptance is becoming more important, especially when corn availability or price fluctuations create challenges. These findings support diversifying cereal sources as a strategy to improve feed security and lessen dependence on corn-based formulations.

Fibrolytic enzyme supplementation in barley- and wheat-based diets modulates gut viscosity, enhances microbial fermentation profiles, and improves nutrient digestibility, thereby maintaining growth performance even in NSP-rich cereals [6, 7, 27]. These mechanisms align with the current findings and support the role of xylanase–β-glucanase complexes in overcoming the antinutritive barriers caused by soluble NSP.

### Study originality and integrated assessment

This study demonstrates originality by combining growth performance, carcass traits, meat physicochemical properties, and consumer sensory evaluation within a single controlled experiment. Unlike prior studies that looked at cereal sources separately, this research directly compares corn, wheat, and barley under isocaloric and isonitrogenous diets supplemented with NSP-degrading enzymes, reducing nutritional confounding. This integrated method offers clearer insight into how cereal traits, like carotenoid content, starch structure, and NSP composition, impact broiler performance and meat quality.

### Practical implications and limitations

The findings of this study highlight the potential to partially replace corn with wheat or barley in broiler diets without compromising growth performance, carcass yield, meat quality, or consumer acceptance. Wheat inclusion may be especially useful for reducing abdominal fat, while corn remains better at increasing meat yellowness, which is preferred in certain markets. Although the feed cost per kilogram varied little among treatments, the similar FCG indicates that adding wheat and barley can be economically feasible, especially in areas where corn supply or prices fluctuate. This underscores the broader sustainability of diversifying cereal sources in poultry nutrition.

However, several limitations should be acknowledged. This study was conducted using only male ROSS 308 broilers reared under controlled environmental conditions, which may not fully reflect the variability of commercial production. Enzyme inclusion was standardized across diets and not evaluated in a dose–response manner. Future research should investigate mixed-sex flocks, higher barley inclusion levels beyond 15%, and dose–response effects of individual NSP-degrading enzyme components. Studies conducted under field conditions with different broiler genotypes would further strengthen the applicability of these findings.

## CONCLUSION

This study showed that partially replacing corn with wheat or barley in enzyme-supplemented, isocaloric, and isonitrogenous broiler diets does not harm overall production performance, carcass yield, meat physicochemical properties, or consumer sensory acceptance. Corn-fed broilers generally gained more weight during the grower and finisher phases, while wheat and barley inclusion maintained similar feed intake, feed efficiency, and mortality rates. Barley-fed broilers had a significantly higher abdominal fat percentage, whereas wheat inclusion resulted in the lowest abdominal fat deposition. Meat yellowness (b*) was highest in corn-fed broilers due to its higher carotenoid content, while wheat- and barley-based diets produced paler breast meat without negatively affecting other meat qualities like pH, water-holding capacity, texture, or cooking loss. Notably, consumer sensory scores for all dietary groups were above 4.3 on a 7-point scale, showing that cereal source did not negatively impact perceived meat quality.

A major strength of this work is its integrated, controlled assessment of growth performance, carcass traits, meat quality parameters, and consumer perceptions within one experimental framework. The use of isocaloric, isonitrogenous diets supplemented with a standardized xylanase–β-glucanase complex reduces confounding nutritional variables and enables direct comparison of cereal-specific effects. Additionally, including consumer sensory evaluation offers valuable insights into market-relevant traits that many nutritional studies often overlook.

Future research should investigate cereal substitution strategies in mixed-sex flocks under commercial field conditions, using broiler genotypes beyond ROSS 308. Examining higher barley inclusion levels, dose–response relationships for individual NSP-degrading enzymes, and how cereal type interacts with gut microbiome composition will provide deeper insights into optimizing nutrient use. Adding economic modeling and environmental impact assessments can also help quantify the sustainability benefits of diversifying cereal sources in broiler production.

Overall, the findings confirm that wheat and barley can serve as practical and economically viable alternatives to corn in modern broiler diets when appropriately formulated and supplemented with NSP-degrading enzymes. Wheat may be strategically used to reduce carcass fat levels, while corn remains advantageous in markets that prefer yellow-pigmented meat. As feed cost volatility and cereal supply fluctuations increase globally, adopting flexible cereal inclusion strategies supported by enzyme technology can boost feed security, production efficiency, and sustainability in broiler production systems.

## DATA AVAILABILITY

All the generated data are included in the manuscript.

## AUTHORS’ CONTRIBUTIONS

TP and PK1: Conceived and designed the study. SP and CR: Conducted the feeding trial. PK1 and SP: Performed laboratory analyses. PK2, TP, and KS: Conducted data analysis. TP, PK1, PK2, and KS: Drafted and revised the manuscript. All authors have read and approved the final version of the manuscript.
